# Predictors of patients’ choice of hospitals under universal health coverage: a case study of the Nicaraguan capital

**DOI:** 10.1186/s12913-021-07333-z

**Published:** 2021-12-19

**Authors:** Ida G. Monfared, Jonathan Garcia, Sebastian Vollmer

**Affiliations:** Centre for Modern Indian Studies & Department of Economics, University of Göttingen, Waldweg 26, 37073 Göttingen, Germany

**Keywords:** Hospital access, Latin America, Nicaragua, Patients’ choice, Referral system

## Abstract

**Background:**

This study looks at the factors that can shape patients’ choice of healthcare providers. Understanding this process can help with making high quality healthcare more accessible for all. We focus on distance, patient’s health status, (perceived) quality of healthcare facility, and referrals to investigate how these factors compete in shaping patients’ choice of hospitals.

**Methods:**

This study was carried out in Managua, the capital of Nicaragua. Utilizing an exit-survey, patients were interviewed across five public hospitals in 2017 and then six in 2019 when a new highly-equipped hospital was added to the system. We used a multinomial logit model to investigate patients’ preference of a specific hospital over the rest within each wave.

**Results:**

Our results show that being referred to a hospital is the strongest predictor and in some cases, it can increase the relative risk ratio of choosing a facility by a factor of 49 (*p* < 0.01; 95% CI: 27.39–87.17). For the remaining factors, the hierarchy of importance was less clear-cut yet all these factors remained significantly important at various levels.

**Conclusions:**

Overall, our results highlight the importance of referral systems in making quality healthcare more equitable. Moreover, with distance also being a key predictor and in the absence of an organized referral system, those with low-income would either be further deprived by having to settle with locally available healthcare (regardless of its quality) or face high amounts of out-of-pocket expenditure when seeking help from the private sector.

**Supplementary Information:**

The online version contains supplementary material available at 10.1186/s12913-021-07333-z.

## Background

Having access to quality healthcare has long been recognized as a basic human right [1]. However, this remains a challenge given the scarcity of resources particularly in low- and middle-income countries (LMICs). It was under the notion of human rights that Latin American countries (LACs) launched a major healthcare system reform aiming to achieve universal health coverage (UHC) [2]. In general, UHC means that all individuals should have access to health services without encountering any financial hardship. With high disparities in implementation of UHC between high-income countries (HICs) and LMICs [3], UHC does not necessarily guarantee quality [4], organizational coordination (i.e. adequate referrals), or choice of service provider for the poor [5]. A notable reform in LACs was the decentralization of healthcare systems that aimed to increase healthcare equity by giving the decision-making power to the local governors [6]. The outcome of this reform, however, was rather mixed. For example, decentralization gave further autonomy to primary and community level healthcare providers that alongside global programs such as Integrated Management of Childhood Illness achieved significant decreases in infant mortality, particularly in hard-to-reach areas [7]. It was also under this reform that the government of Nicaragua, the country setting of the present study, separated the administrative and budgetary systems of primary and secondary care. However, Birn et al. note that this structural change had an adverse impact and led to a breakdown of the referral system, loss of accountability, and reduction in primary care funding [8]. In Nicaragua, the Ministry of Health (MINSA) holds control and is responsible for ensuring healthcare equity under the country’s General Health Law that was signed in 2002 [9]. Prior to this law and during the 1990s, pressure from international financial institutions pushed the government to create parallel private healthcare services without proper consultation or previous experience. In effect, MINSA’s survival became dependent on its support for privatized services, leading to further inequality in provision of healthcare for all [10–12]. In order to mitigate the impact from these reforms, in 2006 the government introduced a new Family and Community Health Care Model to increase healthcare coverage that placed further focus on citizens and community participation [13]. Although these efforts made some services (particularly child and maternal care) more accessible to rural communities [14], they did not resolve the financial and administrative challenges within the wider system [15].

Considering that seeking secondary healthcare through private services could entail substantial costs, the choice of hospital for those with low-income effectively remains limited to the subsidized care provided by public hospitals. Based on evidence from Managua, the capital of Nicaragua, our study aims to determine how under such conditions, various potential factors predict patients process of choice and shape their pathways for accessing secondary healthcare. In particular, the inauguration of a new public referral hospital in Managua presented a rare opportunity to study how patients chose between public hospitals after an additional service became available. This study not only adds to the scarce evidence from a low-income setting in LACs, but also relates to other contexts where the poor have to make similar choices.

Studies of patients’ choice vary widely depending on framework (conceptual [16, 17] or empirical [18]), setting (LMICs [19] or HICs [20–22], scale (multi-country [23] or country level [24]), and service type (primary [25] or secondary [26]). Despite these variations, a number of shared key factors can be identified among the empirical studies carried out in low-income settings. These factors include perceived quality of care, proximity of healthcare facility or transport convenience, perceived morbidity of illness or health status, referral, and costs [18, 27–30]. It is also important to note that the interplay between *choice* and *access* is rather complex and choice is only meaningful when there are options to choose from. Moreover, patients do not always choose their healthcare provider following a systematic comparison of information in order to identify the best quality. Instead, they often choose according to a combination of factors based on their own judgment (e.g. expectation and perception) as well as provider characteristics such as distance and costs [31]. A qualitative study of patients’ choice of hospitals in Iran found that patients often make a judgment call; when the illness is not severe, patients tend to prioritise first by costs and then by proximity. However, in severe cases, both cost and distance become secondary and quality becomes the main priority [32]. Similar observations were made by a study in rural India, which found that when patient health status is poor, distance plays a less significant role in their choice of provider [33]. Severe health conditions also call for professionals appropriate judgment and timely referral. In such cases, referred patients do not choose which health care facility to go to but are advised to go to a specific facility that meets their needs. However, evidence suggests that patients would bypass the referral system when they do not have confidence in the system or when they are given inadequate information about the referral process [34–36].

In Nicaragua, receiving treatment at public healthcare facilities does not entail any costs, hence, this element would not be a primary factor in patients’ process of decision making when choosing between public hospitals. However, the question remains that in such a setting, how the remaining factors, namely distance, (perceived) quality of care, referrals, and health status predict patients’ choice of healthcare facilities.

### Setting

The present study is part of an impact evaluation of the project ORIO10/NI/21 [37], construction of a new hospital (Hospital Occidental De Managua “Dr Fernando Vélez Paiz” (HFV)) in western Managua. In Nicaragua, healthcare coverage is provided via three regimes; contributory which is implemented by the Institute Nicaraguan Social Security (INSS) where contributors and beneficiaries are those who are in official employment and their families (covering about 10% of the population), non-contributory, free UHC for the uninsured (covering more than 70% of the population), and voluntary, which includes private insurance providers (a relatively small sector) [38]. The healthcare system is managed at three administrative levels: first is the central level, second is SILAIS (the Comprehensive Local Health Systems) which are in charge of national reference and departmental hospitals, and third is the municipality level that looks after health centres, health posts, and community-based clinics. Primary care is provided by health centres which are usually staffed with general practitioners or nurses. There is one health centre in each municipality and patients can only visit the health centre that is allocated to their residential area. Under the jurisdiction of health centres are the community-based health network (comprising volunteers or “brigadista” and midwives) and health posts (with one or two nurses and in some cases a doctor) which provide primary and preventive care particularly in rural areas. While patients cannot choose between health centres, they face no barriers in visiting a public hospital of their choice and there are no gatekeepers. Of 31 hospitals managed by MINSA, there are 11 national referral hospitals located in Managua that provide secondary and specialist care [39]. Patients are referred to secondary or tertiary care by general practitioners and family medicine specialists. The system also allows inter-institutional referral although there is no centrally managed electronic system and referrals are solely paper based [40].

In 2018, of around 6.5 million people living in Nicaragua, 50.7% were female, 30.2% were aged 14 or younger, 58.5% lived in urban areas, 30.6% were employed in agriculture, 16.9% in industry, and 52.7% in the service sector [41]. With a growing trend of immigration from rural to urban areas, Managua is densely populated and in 2018 accommodated 24% of the country’s total population [39]. Nicaragua has been through decades of political unrest that have affected the country’s overall development [42]. Moreover, Managua’s infrastructure has been particularly damaged on multiple occasions by devastating earthquakes notably in 1972 that led to over 11,000 deaths and left 75% of houses destroyed [43]. Another earthquake in 2014 put the Mother and Child Hospital Vélez Paiz out of service, which was replaced in 2018 by the HFV. The new hospital provides a wide range of services aiming to reduce the burden on the overstretched existing facilities in Managua.

## Methods

We selected five national referral hospitals that provide similar services as the new hospital while the remainder provide services relating to specific specialties (e.g. mental health) that are not comparable with HFV. The data for this study was collected through patient exit-interviews at these hospitals in 2017, prior to the construction of the new hospital and then included HFV in 2019. Patients leaving these hospitals were randomly selected and invited to voluntarily participate in the survey if they met the following inclusion criteria: they were at least 16 years old, capable of consenting to and participating in the interview, were themselves a patient or accompanied a patient (e.g. parents), had already been attended by a doctor or nurse during their current visit, and were finished with their visit/stay at the health facility for the day. In addition to basic information on patients’ background including their place of residence, the survey measured a wide-range of aspects related to patient’s experiences. Enumerators were locally recruited through the Centre for Research and Health Studies in Managua (CIES-UNAN Managua) and were trained and supervised by the research team.

The primary sample size was determined as part of the wider study of the impact evaluation of construction of the new hospital with its findings to be disseminated in the near future (please find further details in Additional file 1: Appendix A section I). For the purpose of the present study, we used a multinomial logit model (MNLM) where the study outcome “hospital choice” is a categorical variable with no possibility of overlaps between options. 

The MNLM equation (1) gives the relative probability *β* of a patient choosing hospital *m* as opposed to another health facility *b* (known as reference) [44]:

$$\ln {\Omega}_{m\mid b}\left(\boldsymbol{x}\right)=\ln \frac{\Pr \left(y=m\ |\ \boldsymbol{x}\right)}{\Pr \left(y=b\ |\ \boldsymbol{x}\right)}=\boldsymbol{x}\ {\beta}_{m\mid b}\kern4.75em (1)$$

where *m* = 1 to *K* (*K* being the total number of available public hospitals in Managua in each wave). Data on potential covariates such as patient’s age, gender, years of schooling, insurance status, chronic illness, waiting time, and self-reported out-of-pocket (OoP) expenditure within last 30 days on travel and on medication were collected during the patient interviews and were included in the model (the complete list of these variables is presented in Additional file 1: Appendix A, Section II). We ran this regression for each wave in 2017 (pre-) and 2019 (post-construction of HFV) to investigate which aspects were the predictors of choice. As an indicator of (perceived) quality of care, we built a single indicator for patients’ global rating of the health facility based on polychoric factor analysis [45] that included patients’ response to their overall satisfaction with the service they received (5-point scale), overall rating of the health facility (11-point scale), recommendation to friends and family (4-point scale), and amount of improvement they think the health facility needs (4-point scale) with higher scores indicating better ratings (please see Additional file 1: Appendix A, Section III). Stata 14.2 was used to run the analysis and QGIS 3.14 for calculating the distance and creating maps.

## Results

Table 1 presents patients' characteristics and the number of interviews carried out in each wave (see Table A.1. in Additional file 1: Appendix A). Here, health status is measured on a 4-point scale (1 = *very good* to 4 = *poor*).

**Table 1:** Summary characteristics of patients who were interviewed in each wave

	2017 (*N* = 1934)				2019 (*N* = 2431)			
	Mean / Ratio	SD.	Min	Max	Mean / Ratio	SD.	Min	Max
Age	38.79	23.85	0	96	33.86	22.60	0	98
Female (%)	69.44				67.26			
Years of schooling	8.01	4.42	0	22	8.65	4.39	0	18
Employed (%)	24.30				29.68			
Uninsured (non-contributory regime) (%)	82.73				89.51			
Health status	2.53	0.73	1	4	2.52	0.83	1	4
Global rating	5.34	0.98	0.76	6.59	5.50	0.83	0.76	6.59

In both years, the majority of patients were female, the average age was in the 30s, and years of education were similar. Also, in both waves, the share of uninsured patients was noticeably high. Table 2 shows the frequencies of patients’ responses to the question as to why they chose to visit each health facility across waves.

**Table 2:** Patients self-reported reason for choice of each hospital in 2017 and 2019 (%)

Hospitals	Distance		Quality of service		Always came here		Recommend by Friend / family		Referred by another medical centre		Have a friend / family who works here	
	2017	2019	2017	2019	2017	2019	2017	2019	2017	2019	2017	2019
H1	2.66	1.82	3.46	1.3	30.05	0.78	2.39	0.78	59.84	92.45	1.6	2.86
H2	8.83	4.48	40.52	51.99	8.31	13.43	1.56	3.48	39.48	25.12	1.3	1.49
H3	0.26	2.94	47.23	52.7	3.43	1.23	0.79	3.68	46.17	37.25	2.11	2.21
H4	27.04	38.01	26.02	19.85	14.8	4.84	1.79	0.24	27.55	35.84	2.81	1.21
H5	0	0	50.14	14.48	2.45	0	0.27	4.56	46.32	80.7	0.82	0.27
HFV	–	25.92	–	39.61	–	9.05	–	10.76	–	13.2	–	1.47
Total (overall facilities)	7.95	12.56	33.39	30.43	11.85	4.98	1.37	3.93	43.71	46.5	1.74	1.59

Although the reasons patients gave for their choice varies considerably between hospitals, overall, being referred by another medical centre and the quality of service are the two most frequently provided reasons. H3 and H5 are specialist hospitals, which could explain why distance was not given as a reason for visiting these hospitals. We used this self-reported reason to create a dummy variable which equals 1 if patients reported that they were referred, and 0 otherwise. This variable was then used in our MNLM (Eq. [1]) as a predictor along with the objective geographical distance between patients’ place of residence and the health facility they visited, as well as patients’ global rating of the facility to learn how these factors compete (please see further details on the variables definition and manipulation in the Additional file 1: Appendix A, Section I, Table A.2).

**Fig. 1:**
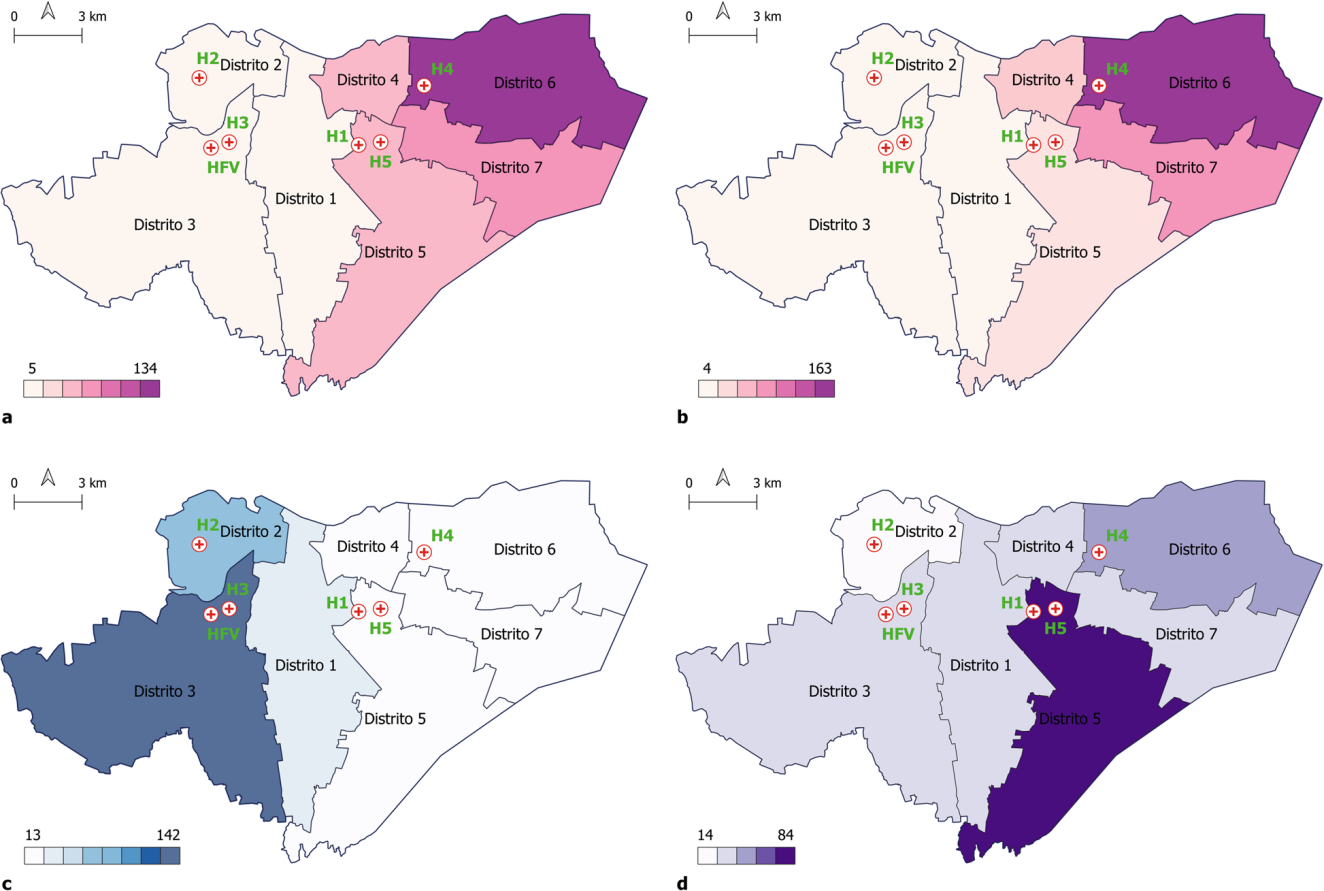
Distribution of patients’ place of residence that visited H4 in 2017 (**a**) compared to those visiting the same facility in 2019 (**b**). Figure (**c**) illustrates this distribution for those visiting the new hospital HFV in 2019 compared to those who visited H1 in the same year (**d**) (district borders are adapted from© 2017 mapanica.net)

Figure 1  (a and b) shows the place of residence of patients who visited the hospital located in the East side of the city, H4, before and after the construction of the new hospital. This hospital is chosen as it is farthest away from HFV. At the first glance, it seems that the construction of HFV had no impact on the choice for patients living in the East side of the city, and for those visiting H4 distance might be a more important factor than seeking healthcare from a newly constructed hospital. Figure 1 (c and d) illustrates the place of residence of patients visiting HFV and H1 in 2019, respectively. Those who visited the new hospital seem to be mostly residing in the West side of the city with distance as the key predictor. A noticeable contrast, however, is hospital H1 which had the highest rate of referral patients (Table 2). As Fig. 1(c) illustrates, place of residence for patients who visited this hospital was more scattered covering a wider area of the city indicating that the competition between referral and distance as key predictors might be less clear cut.

Table 3 shows the results of estimating Eq. [1] for 2019 with HFV as reference. It presents the relative risk ratios (*rrr*) of selecting a hospital (each column) as opposed to choosing the new hospital. Here, various potential covariates such as patient’s age, gender, insurance status, waiting time, travel time, OoP expenditure (last 30 days) on travel, OoP on medication, and visiting due to a chronic illness are controlled for (see Table A.3 in Additional file 1: Appendix A for full list of covariates).

**Table 3:** Relative risk ratio of patients choosing another hospital over the new hospital in 2019 (*N* = 1912)

	H1	H2	H3	H4	H5
Distance (log km)	1.77*** (1.39–2.27)	1.17 (0.95–1.45)	1.26** (1.00–1.57)	0.78** (0.62–0.98)	1.62** (1.11–2.35)
Being referred	48.86*** (27.39–87.17)	1.44 (0.89–2.30)	1.73** (1.07–2.81)	3.80*** (2.42–5.97)	36.93*** (18.18–75.03)
Health status	0.76** (0.58–0.99)	0.88 (0.70–1.10)	0.28*** (0.22–0.36)	0.65*** (0.52–0.81)	2.53*** (1.63–3.93)
Global rating	0.82 (0.61–1.09)	0.73** (0.57–0.93)	0.56*** (0.43–0.73)	0.43*** (0.35–0.54)	1.03 (0.69–1.54)

95% CI in parentheses; *** *p* < 0.01, ** *p* < 0.05, * *p* < 0.1

It does not come as a surprise to see that the distance between patients’ place of residence and the hospital is an important factor. However, being referred stands out particularly in the case of choosing hospitals H1 and H5 over the new hospital. In both cases, patients rating of hospital quality is no longer significant. On the other hand, with one unit increase in perceived quality, the relative risk of choosing hospitals H2, H3, and H4 over the new hospital decreases significantly. In the case of H4, being referred increases the relative risk of choosing this hospital instead of HFV by a factor of 3.8 ceteris paribus.

In order to see whether or not this pattern changed over time, we ran the analysis for each wave placing H4 as the reference as this hospital is farthest away from the new hospital (Table 4).

**Table 4:** Relative risk ratio of patients choosing another hospital over H4 in each wave

	2017 (*N* = 1629)				2019 (*N* = 1912)				
	H1	H2	H3	H5	H1	H2	H3	H5	HFV
Distance (log km)	1.53*** (1.24–1.89)	1.46*** (1.20–1.79)	1.41*** (1.15–1.71)	1.92*** (1.38–2.68)	2.27*** (1.76–2.92)	1.50*** (1.20–1.88)	1.61*** (1.27–2.03)	2.07*** (1.41–3.04)	1.28** (1.02–1.61)
Being referred	2.98*** (2.04–4.35)	1.53** (1.06–2.22)	1.55** (1.08–2.22)	2.66*** (1.42–4.96)	12.86*** (7.54–21.93)	0.38*** (0.25–0.58)	0.46*** (0.30–0.70)	9.72*** (4.87–19.40)	0.26*** (0.17–0.41)
Health status	0.67*** (0.52–0.87)	1.44*** (1.13–1.83)	0.75** (0.58–0.96)	2.64*** (1.67–4.17)	1.17 (0.91–1.52)	1.35** (1.07–1.71)	0.43*** (0.33–0.55)	3.90*** (2.49–6.09)	1.54*** (1.23–1.93)
Global rating	0.61*** (0.50–0.73)	1.30*** (1.07–1.58)	0.97 (0.79–1.17)	1.26 (0.90–1.77)	1.88*** (1.48–2.40)	1.67*** (1.36–2.06)	1.29** (1.03–1.61)	2.38*** (1.63–3.47)	2.31*** (1.84–2.89)

95% CI in parentheses; *** *p* < 0.01, ** *p* < 0.05, * *p* < 0.1

Again, in some of the cases being referred to a facility can be a major predictor of choice and this becomes more apparent in the second wave as the ratio of referrals visiting H1 and H5 in 2019 increases. The other domains of health status and perceived quality remain significant. The largest factor for patients choosing the new hospital over H4 is the new hospital’s quality. In other cases, it can be seen that besides the referral, the order of importance slightly changes over time.

We also found that the relative risk of choosing H5 over H4 in 2017 increased by a factor of 3.3 for those who had some form of insurance compared to those who had none (*p* < 0.01; 95% CI: 1.53–6.89). That was in a similar scale of 3.4 in 2019 (*p* < 0.05; 95% CI: 1.23–9.35). Having insurance also increases the relative risk of choosing H5 over the new hospital to a similar extent (*rrr* = 3.2; *p* < 0.05; 95% CI: 1.17–8.49). Being referred increased the relative risk of choosing H5 over the new hospital far more (*rrr* = 36.93 *p* < 0.01; 95% CI: 18.18–75.03).

As a robustness check, we repeated the analysis using H2 as the reference and found again that although distance and perceived quality are significant factors in choosing other hospitals over H2, being referred remains a strong predictor (see Additional file 1: Appendix A, Section IV).

## Discussion

The present study illustrates how, in a setting where resources are scarce, key factors such as (perceived) quality of care, patients’ health status, distance to the care provider, and referrals compete in shaping patients’ choice of a healthcare facility. The results indicate that when patients are referred to a hospital, this becomes the strongest predictor. After referrals distance seems to be a key predictor of patients’ choice of hospital in almost all cases. This is in line with observations from other studies in LMICs where patients noted proximity to be very important [29, 30]. Similar to evidence from previous studies, we find that perceived quality and patient’s health status to be also important factors [18, 27]. Overall, our results indicate that in each instance, patients make a judgment call based on their conditions and perceived information balancing between all these factors to make a choice. These findings support a subtlety in patients’ decision making that was also noted by previous qualitative studies [32].

Our study highlights the importance of adequacy of the referrals system. A stark contrast can be seen between the distribution of place of residence of patients who visited H4 as well as the new hospital as opposed to those who visited H1. While primarily in the former cases the distance appears to be a defining element, a glance at the latter indicates that when the rate of referrals is high, the hospital would cover a wider geographical area. This highlights how a substantial investment in infrastructure, here construction of a new public hospital, might fail to meet its full potential if it is not properly integrated into the referral system.

Mwabu notes that in LMICs, referral systems and patients care seeking behaviour are often similar in terms of minimizing costs [46]. However, the referral system needs to be efficient and equitable to maintain fair access to quality healthcare. A well-functioning referral system also requires a good level of communication between all the parties involved; lack of coordination within the healthcare system as well as inadequate communication between facilities, healthcare staff, and patients can result in treatment delays, patients bypassing the system [47], and inappropriate use of emergency services [48].

The Nicaraguan healthcare system faces similar challenges as other LACs summarized by Atun et al. [6]; growing wealth disparities, difference in quality of service between the public and private sector, persistent high amounts of OoP expenditure, an aging population (1.7 percentage point increase in the population share of those who are aged 65 years and above within two decades [49]), rapid urbanisation, and fragility of the economy. While UHC was originally introduced to make healthcare services accessible to all, lack of coordination within the system as a result of decentralization has had an adverse impact on the referral system. As noted by Birn et al.*,* SILAIS’s income from selling services to INSS was reinvested only in the same sector rather than being distributed universally [8], creating further inequality in allocation of resources [50]. In recent years, the government has attempted to increase equity by empowering communities [51], which has had some positive impact particularly in remote areas [52]. However, a study carried out by Hartmann in 2018 indicates a remaining gap between the government’s rhetoric of ‘Live Beautiful, Live Well’ (‘Vivir Bonito, Vivir Bien’) campaign launched in 2013 and the reality of living conditions [53].

Moreover, recent political unrest in 2018 had a major impact not only on the country’s economy, but also on its healthcare system as treating those who were injured during the unrest led to prosecutions of healthcare workforce and unjustified dismissals of doctors and nurses [54], which significantly politicised the system and led to uncertainties that are still present [55]. This has added further pressure on already over-stretched resources. In the absence of an adequate referral system, the restrictions imposed by geography and distance could lead to an uneven healthcare system creating postcode lotteries and pockets of poverty where quality healthcare becomes only accessible to those that happen to live in the catchment area of facilities which provide better quality of care. This in turn compel those who cannot access quality healthcare to either settle for a local (albeit possibly inadequate) service or face substantial OoP expenditures by seeking help from the private sector.

### Study limitations

Without access to administrative data our study is limited to findings from patient feedback. In particular, the quality of care is only measured through patients’ perceptions while quality assurance based on evidence from the supply side is also important. Our study also shares similar limitations to other studies that are based on self-reported measures and relies on respondents’ judgment. In addition, the study only covers an urban area where patients could choose between healthcare facilities. Nonetheless, this work presents observations from a region where evidence is rather limited.

## Conclusion

Our study investigated how various factors that shape patients’ decision making in choosing a hospital compete. It was observed that patients being referred to a facility was an outstanding factor in determining hospital choice. The findings highlight that the quality of referral systems, particularly in a region with scarce resources, is of utmost importance. We found less clear-cut results for the reaming factors that are distance, patient’s health status, and (perceived) quality of healthcare facility. It appears that patients’ choice is based on a combination of various factors that could slightly change in strength depending on time and occasions, yet all of these factors remain important. To ensure equitable access to quality healthcare, it is essential to have an adequate referral system. Moreover, quality care should be made available to all regardless of their place of residence. 

## Supplementary Information

**Additional file 1.**

## Data Availability

The datasets generated and/or analysed during the current study are not publicly available due to the other publications from the study still in progress but are available from the corresponding author on reasonable request.

## Abbreviations

LMICs: Low- and Middle-income Countries
MINSA: Nicaraguan Ministry of Health
WHO: the World Health Organization
OoP: Out-of-Pocket
HFV: Hospital Occidental De Managua “Dr Fernando Vélez Paiz”
HICs: High-income Countries
INSS: the Institute Nicaraguan Social Security
LACs: Latin American Countries
SILAIS: the Comprehensive Local Health Systems
CIES-UNAN Managua: Centre for Research and Health Studies in Managua
MNLM: Multinomial Logit Model
UHC: Universal Health Coverage
