# Supplementary material for: Predictors of patients’ choice of hospitals under universal health coverage: a case study of the Nicaraguan capital
Source: BMC Health Serv Res. 2021 Dec 19;21:1356. doi: 10.1186/s12913-021-07333-z (PMC8684609; doi:10.1186/s12913-021-07333-z)
Supplement: Supplementary file 1 — Additional file 1. [file 12913_2021_7333_MOESM1_ESM.docx]

# Appendix A

**A.I. Sample size, demographics and covariates details**

The present study is part of the wider project “impact evaluation of the construction of the new Hospital Occidental De Managua Dr Fernando Vélez Paiz” (ORIO10/NI/21^[[1]](#footnote-1)^) assessing the impact of this project on the performance of existing public healthcare services in Managua. The sample size for the study was determined at the baseline with particular focus placed on patients’ perceived quality of care^[[2]](#footnote-2)^ and waiting time^[[3]](#footnote-3)^ that are also contributing factors in patients’ process of choice. The power calculations indicated that the sample size of 1904 cases per each wave would suffice to detect minimum effect with 90% power. Table A.1. presents the number of interviews carried out at each hospital during each wave.

| **Table A.1.** Number of interviews carried out at each hospital site within each wave (year). | | | | | | | |
| --- | --- | --- | --- | --- | --- | --- | --- |
| Wave | H1 | H2 | H3 | H4 | H5 | HFV | Total |
| 2017 | 380 | 396 | 393 | 395 | 370 | 0 | 1,934 |
| 2019 | 406 | 408 | 408 | 418 | 374 | 417 | 2,431 |
| Total | 786 | 804 | 801 | 813 | 744 | 417 | 4,365 |

For the purpose of the present study a Multinomial Logit Model (MNLM) was used for which a minimum of 10 observations per each group is recommended^[[4]](#footnote-4)^. Our sample size exceeds this requirement with the exception of the gender groups for maternity hospital, hence, figures for this factor are not reported.

Table A.2. presents further details on demographics and other covariates definition and manipulation. Data for all these variables were collected during the interview with patients as they were leaving the hospitals. The distance between the patients’ place of residence and the health facility is calculated using the software QGIS 3.14.

| **Table A.2.** The description of demographics and other covariates used in the study model. Distance was calculated objectively between patients’ place of residence (self-declared) and the location of the hospital they visited. The rest of the variables were collected during the interview with patients and are self-reported. | |
| --- | --- |
| Variable | Definition |
| Distance | The distance between patient’s place of residence at the district level and the health facility they visited in log Kilometers. |
| Being referred | Dummy variable = 1 if patient was referred, otherwise = 0. |
| Female | Dummy variable = 1 if patient was female, otherwise = 0. |
| Age | Measured in years. |
| Employment | Dummy variable = 1 if patient was employed (either official or self-employed), otherwise = 0. |
| Schooling | Total year of education completed. |
| Insurance status | Dummy variable = 1 if patient had any type of insurance including INSS and private, otherwise = 0 (uninsured). |
| Health status | 4-point scale with 1 = Very good to 4 = Poor. |
| Waiting time | The time between when patient arrived and when they were attended in log minutes. |
| Travel time | The typical average time that usually took patient to get to the present hospital from home, at an average day of the week around 10am (self-reported) in log minutes. |
| OoP expenditure on transport | Amount spent in the past 30 days on transport to a health care service (self-reported) in log córdoba. |
| OoP expenditure on medicine | Amount spent in the past 30 days on medicine and other things related to health care such as alcohol, cotton, gauze etc. (self-reported) in log córdoba. |
| Chronic | Dummy variable = 1 if the illness due to which the present visit was made was chronic, otherwise = 0. |

## A.II. Full list of covariates

Table A.3. presents the results for choosing another hospital over the new hospital in 2019 with full list of covariates presented.

| **Table A.3.** Relative risk ratio of patients choosing another hospital over the new hospital in 2019 (*N* = 1,912). | | | | | |
| --- | --- | --- | --- | --- | --- |
|  | H1 | H2 | H3 | H4 | H5 |
| Distance (log km) | 1.77*** | 1.17 | 1.26** | 0.78** | 1.62** |
|  | (1.39 - 2.27) | (0.95 - 1.45) | (1.00 - 1.57) | (0.62 - 0.98) | (1.11 - 2.35) |
| Being referred | 48.86*** | 1.44 | 1.73** | 3.80*** | 36.93*** |
|  | (27.39 - 87.17) | (0.89 - 2.30) | (1.07 - 2.81) | (2.42 - 5.97) | (18.18 - 75.03) |
| Global rating | 0.82 | 0.73** | 0.56*** | 0.43*** | 1.03 |
|  | (0.61 - 1.09) | (0.57 - 0.93) | (0.43 - 0.73) | (0.35 - 0.54) | (0.69 - 1.54) |
| Female | 0.54** | 0.38*** | *error*^✝^ | 1.29 | 1.27 |
|  | (0.33 - 0.87) | (0.26 - 0.56) |  | (0.86 - 1.94) | (0.70 - 2.33) |
| Age in years | 1.06*** | 1.06*** | 1.05*** | 1.02*** | 0.78*** |
|  | (1.05 - 1.08) | (1.04 - 1.07) | (1.04 - 1.06) | (1.01 - 1.03) | (0.74 - 0.82) |
| Employment dummy (1-yes) | 0.70 | 1.57** | 0.91 | 0.94 | 0.85 |
|  | (0.44 - 1.14) | (1.08 - 2.31) | (0.59 - 1.42) | (0.64 - 1.38) | (0.44 - 1.64) |
| Schooling | 1.00 | 1.04 | 1.00 | 0.99 | 1.09** |
|  | (0.95 - 1.06) | (0.99 - 1.08) | (0.95 - 1.05) | (0.95 - 1.04) | (1.00 - 1.18) |
| Insurance dummy (1-yes) | 1.95* | 0.62 | 0.25*** | 0.93 | 3.15** |
|  | (0.94 - 4.04) | (0.32 - 1.20) | (0.10 - 0.62) | (0.48 - 1.80) | (1.17 - 8.49) |
| Health status (1 = very good to 4 = poor) | 0.76** | 0.88 | 0.28*** | 0.65*** | 2.53*** |
|  | (0.58 - 0.99) | (0.70 - 1.10) | (0.22 - 0.36) | (0.52 - 0.81) | (1.63 - 3.93) |
| Waiting time (log minutes) | 2.19*** | 1.45*** | 1.35*** | 1.29*** | 3.00*** |
|  | (1.74 - 2.74) | (1.21 - 1.73) | (1.11 - 1.63) | (1.09 - 1.53) | (2.20 - 4.10) |
| Travel time (log minutes) | 0.99 | 1.84*** | 1.92*** | 1.04 | 2.44*** |
|  | (0.70 - 1.39) | (1.36 - 2.50) | (1.38 - 2.67) | (0.77 - 1.39) | (1.43 - 4.17) |
| OoP expenditure on transport (log córdoba) | 0.88* | 1.04 | 1.31*** | 0.93 | 1.09 |
|  | (0.76 - 1.02) | (0.92 - 1.18) | (1.14 - 1.51) | (0.83 - 1.04) | (0.86 - 1.39) |
| OoP expenditure on medicine (log córdoba) | 0.82*** | 1.09*** | 0.83*** | 1.04 | 0.75*** |
|  | (0.76 - 0.89) | (1.03 - 1.16) | (0.77 - 0.89) | (0.98 - 1.11) | (0.67 - 0.84) |
| Chronic (1-yes) | 0.27*** | 0.70* | 0.33*** | 0.52*** | 0.70 |
|  | (0.17 - 0.43) | (0.47 - 1.05) | (0.21 - 0.51) | (0.34 - 0.79) | (0.35 - 1.39) |
| 95% CI in parentheses; *** p<0.01, ** p<0.05, * p<0.1.  ^✝^ H3 is a specialist maternity hospital; hence, being female returned unusually large coefficients in this instance. | | | | | |

Table A.4. shows the results for choosing another hospital over H4 in 2017 and 2019 with full list of covariates presented.

| \| **Table A.4.** Relative risk ratio of patients choosing another hospital over H4 in each wave. \| \| --- \| | | | | | | | | | |  |
| --- | --- | --- | --- | --- | --- | --- | --- | --- | --- | --- | --- |
|  | 2017 (*N* = 1,629 ) | | | | 2019 (*N* = 1,912) | | | | |  |
|  | H1 | H2 | H3 | H5 | H1 | H2 | H3 | H5 | HFV | |
| Distance (log km) | 1.53*** | 1.46*** | 1.41*** | 1.92*** | 2.27*** | 1.50*** | 1.61*** | 2.07*** | 1.28** | |
|  | (1.24 - 1.89) | (1.20 - 1.79) | (1.15 - 1.71) | (1.38 - 2.68) | (1.76 - 2.92) | (1.20 - 1.88) | (1.27 - 2.03) | (1.41 - 3.04) | (1.02 - 1.61) | |
| Being referred | 2.98*** | 1.53** | 1.55** | 2.66*** | 12.86*** | 0.38*** | 0.46*** | 9.72*** | 0.26*** | |
|  | (2.04 - 4.35) | (1.06 - 2.22) | (1.08 - 2.22) | (1.42 - 4.96) | (7.54 - 21.93) | (0.25 - 0.58) | (0.30 - 0.70) | (4.87 - 19.40) | (0.17 - 0.41) | |
| Global rating | 0.61*** | 1.30*** | 0.97 | 1.26 | 1.88*** | 1.67*** | 1.29** | 2.38*** | 2.31*** | |
|  | (0.50 - 0.73) | (1.07 - 1.58) | (0.79 - 1.17) | (0.90 - 1.77) | (1.48 - 2.40) | (1.36 - 2.06) | (1.03 - 1.61) | (1.63 - 3.47) | (1.84 - 2.89) | |
| Female | 0.65** | 0.52*** | *error*^✝^ | 0.91 | 0.41*** | 0.29*** | *error*^✝^ | 0.99 | 0.77 | |
|  | (0.43 - 0.99) | (0.35 - 0.76) |  | (0.51 - 1.61) | (0.26 - 0.67) | (0.19 - 0.45) |  | (0.53 - 1.84) | (0.52 - 1.16) | |
| Age in years | 1.05*** | 1.03*** | 1.02*** | 0.77*** | 1.04*** | 1.04*** | 1.03*** | 0.77*** | 0.98*** | |
|  | (1.04 - 1.07) | (1.02 - 1.04) | (1.01 - 1.03) | (0.74 - 0.81) | (1.03 - 1.06) | (1.03 - 1.05) | (1.02 - 1.04) | (0.73 - 0.81) | (0.97 - 0.99) | |
| Employment dummy (1-yes) | 0.44*** | 1.08 | 0.83 | 0.87 | 0.75 | 1.68** | 0.98 | 0.91 | 1.07 | |
|  | (0.27 - 0.71) | (0.72 - 1.62) | (0.55 - 1.25) | (0.47 - 1.61) | (0.47 - 1.20) | (1.13 - 2.51) | (0.63 - 1.51) | (0.47 - 1.78) | (0.73 - 1.57) | |
| Schooling | 1.05** | 1.07*** | 1.06** | 1.17*** | 1.01 | 1.05* | 1.00 | 1.10** | 1.01 | |
|  | (1.00 - 1.10) | (1.03 - 1.12) | (1.01 - 1.11) | (1.08 - 1.27) | (0.96 - 1.06) | (1.00 - 1.10) | (0.96 - 1.05) | (1.01 - 1.20) | (0.96 - 1.06) | |
| Insurance dummy (1-yes) | 1.04 | 0.56** | 0.75 | 3.25*** | 2.10** | 0.67 | 0.27*** | 3.39** | 1.08 | |
|  | (0.64 - 1.69) | (0.33 - 0.93) | (0.45 - 1.27) | (1.53 - 6.89) | (1.05 - 4.18) | (0.34 - 1.32) | (0.11 - 0.66) | (1.23 - 9.35) | (0.55 - 2.09) | |
| Health status (1 = very good to 4 = poor) | 0.67*** | 1.44*** | 0.75** | 2.64*** | 1.17 | 1.35** | 0.43*** | 3.90*** | 1.54*** | |
|  | (0.52 - 0.87) | (1.13 - 1.83) | (0.58 - 0.96) | (1.67 - 4.17) | (0.91 - 1.52) | (1.07 - 1.71) | (0.33 - 0.55) | (2.49 - 6.09) | (1.23 - 1.93) | |
| Waiting time (log minutes) | 0.92 | 2.80*** | 1.15 | 1.75*** | 1.69*** | 1.12 | 1.04 | 2.33*** | 0.77*** | |
|  | (0.77 - 1.11) | (2.27 - 3.45) | (0.97 - 1.38) | (1.33 - 2.31) | (1.36 - 2.12) | (0.93 - 1.35) | (0.86 - 1.26) | (1.69 - 3.20) | (0.65 - 0.92) | |
| Travel time (log minutes) | 1.14 | 1.49*** | 1.96*** | 1.78** | 0.95 | 1.78*** | 1.85*** | 2.35*** | 0.96 | |
|  | (0.85 - 1.53) | (1.13 - 1.97) | (1.48 - 2.60) | (1.14 - 2.79) | (0.68 - 1.32) | (1.30 - 2.44) | (1.34 - 2.57) | (1.37 - 4.05) | (0.72 - 1.29) | |
| OoP expenditure on transport (log córdoba) | 0.98 | 0.90** | 0.85*** | 1.05 | 0.95 | 1.13* | 1.42*** | 1.18 | 1.08 | |
|  | (0.88 - 1.09) | (0.81 - 0.99) | (0.77 - 0.95) | (0.87 - 1.28) | (0.83 - 1.09) | (1.00 - 1.27) | (1.24 - 1.62) | (0.93 - 1.50) | (0.96 - 1.21) | |
| OoP expenditure on medicine (log córdoba) | 0.95 | 1.07** | 1.04 | 0.93 | 0.79*** | 1.05 | 0.80*** | 0.72*** | 0.96 | |
|  | (0.90 - 1.01) | (1.01 - 1.13) | (0.98 - 1.10) | (0.85 - 1.03) | (0.73 - 0.85) | (0.98 - 1.12) | (0.74 - 0.85) | (0.64 - 0.80) | (0.90 - 1.02) | |
| Chronic (1-yes) | 0.29*** | 0.61*** | 0.31*** | 0.53* | 0.52*** | 1.36 | 0.63** | 1.36 | 1.94*** | |
|  | (0.20 - 0.44) | (0.42 - 0.88) | (0.21 - 0.46) | (0.28 - 1.00) | (0.33 - 0.82) | (0.90 - 2.06) | (0.40 - 0.99) | (0.67 - 2.75) | (1.27 - 2.94) | |
| 95% CI in parentheses; *** p<0.01, ** p<0.05, * p<0.1.  ^✝^ H3 is a specialist maternity hospital; hence, being female returned unusually large coefficients in this instance. | | | | | | | | | | |

## A.III. Construction of an indicator for perceived quality

As an indicator for quality of the health facility perceived by patients, we build an indicator as the facility global rating. For this, we used polychoric factor analysis that included patients’ response to their overall satisfaction with the service they received, overall rating of health facility, whether or not recommending the health facility to friends and family, and amount of improvement that they think the health facility needs where higher scores indicate better ratings. Table A.5. presents the correlation matrix of these variables.

| **Table A.5.** Polychoric correlation matrix of variables used to build a single indicator as Global rating of the facility. | | | | |
| --- | --- | --- | --- | --- |
|  | Improvement needed | Overall rating | Recommend to friends and family | Satisfaction with the service |
| Improvement needed | 1.00 |  |  |  |
| Overall rating | 0.41 | 1.00 |  |  |
| Recommend to friends and family | 0.42 | 0.60 | 1.00 |  |
| Satisfaction with the service | 0.38 | 0.48 | 0.48 | 1.00 |

This process yielded one factor with scoring coefficients presented in Table A.6.

| **Table A.6.** Scoring coefficients (method = regression; based on varimax rotated factors). | |
| --- | --- |
| Improvement needed | 0.18 |
| Overall rating | 0.33 |
| Recommend to friends and family | 0.34 |
| Satisfaction with the service | 0.24 |

**A.IV. Robustness check**

As a robustness check, we repeated the analysis using H2 as the reference. As it can be seen in Table A.7., similar to the previous findings, being referred remain a strong predictor of choice particularly in 2019 and for those who visited e.g. H1. It is also worth it to note that geographically H2, is located on west side of the city and is closer to the new hospital, HFV, than to e.g. H4, hence, distance is not a predictor of choosing between HFV and H2, but it matters when it comes to choosing between H4 and H2.

| \| **Table A.7.** Relative risk ratio of patients choosing another hospital over H2 in each wave. \| \| --- \| | | | | | | | | | |  |
| --- | --- | --- | --- | --- | --- | --- | --- | --- | --- | --- | --- |
|  | 2017 (*N* = 1,629 ) | | | | 2019 (*N* = 1,912) | | | | |  |
|  | H1 | H3 | H4 | H5 | H1 | H3 | H4 | H5 | HFV | |
| Distance (log km) | 1.04 | 0.96 | 0.68*** | 1.31 | 1.51*** | 1.07 | 0.67*** | 1.38* | 0.85 | |
|  | (0.86 - 1.27) | (0.81 - 1.15) | (0.56 - 0.83) | (0.94 - 1.83) | (1.20 - 1.90) | (0.86 - 1.33) | (0.53 - 0.84) | (0.95 - 2.01) | (0.69 - 1.05) | |
| Being referred | 1.94*** | 1.01 | 0.65** | 1.74* | 34.03*** | 1.21 | 2.65*** | 25.72*** | 0.70 | |
|  | (1.34 - 2.82) | (0.71 - 1.44) | (0.45 - 0.94) | (0.92 - 3.27) | (19.97 - 57.97) | (0.78 - 1.87) | (1.74 - 4.03) | (12.60 - 52.51) | (0.43 - 1.12) | |
| Global rating | 0.47*** | 0.74*** | 0.77*** | 0.97 | 1.13 | 0.77** | 0.60*** | 1.42* | 1.38** | |
|  | (0.38 - 0.56) | (0.61 - 0.90) | (0.63 - 0.94) | (0.68 - 1.37) | (0.87 - 1.46) | (0.60 - 0.98) | (0.49 - 0.74) | (0.95 - 2.12) | (1.08 - 1.76) | |
| Female | 1.27 | *error*^✝^ | 1.94*** | 1.76* | 1.41 | *error*^✝^ | 3.39*** | 3.34*** | 2.62*** | |
|  | (0.85 - 1.90) |  | (1.31 - 2.87) | (0.96 - 3.22) | (0.90 - 2.19) |  | (2.24 - 5.13) | (1.79 - 6.25) | (1.78 - 3.88) | |
| Age in years | 1.02*** | 0.99 | 0.97*** | 0.75*** | 1.01 | 0.99 | 0.96*** | 0.74*** | 0.95*** | |
|  | (1.01 - 1.04) | (0.98 - 1.00) | (0.96 - 0.98) | (0.72 - 0.78) | (0.99 - 1.02) | (0.98 - 1.00) | (0.95 - 0.98) | (0.70 - 0.78) | (0.94 - 0.96) | |
| Employment dummy (1-yes) | 0.41*** | 0.76 | 0.92 | 0.80 | 0.45*** | 0.58** | 0.59** | 0.54* | 0.63** | |
|  | (0.25 - 0.66) | (0.50 - 1.17) | (0.62 - 1.38) | (0.42 - 1.54) | (0.28 - 0.71) | (0.38 - 0.90) | (0.40 - 0.89) | (0.28 - 1.06) | (0.43 - 0.93) | |
| Schooling | 0.98 | 0.99 | 0.93*** | 1.09** | 0.97 | 0.96 | 0.96* | 1.05 | 0.97 | |
|  | (0.93 - 1.02) | (0.94 - 1.03) | (0.89 - 0.97) | (1.01 - 1.18) | (0.92 - 1.02) | (0.92 - 1.01) | (0.91 - 1.00) | (0.97 - 1.14) | (0.92 - 1.01) | |
| Insurance dummy (1-yes) | 1.87** | 1.36 | 1.80** | 5.84*** | 3.13*** | 0.40** | 1.49 | 5.06*** | 1.60 | |
|  | (1.11 - 3.13) | (0.78 - 2.36) | (1.08 - 3.01) | (2.60 - 13.15) | (1.57 - 6.23) | (0.16 - 0.98) | (0.76 - 2.93) | (1.79 - 14.25) | (0.83 - 3.10) | |
| Health status (1 = very good to 4 = poor) | 0.47*** | 0.52*** | 0.70*** | 1.84** | 0.87 | 0.32*** | 0.74** | 2.89*** | 1.14 | |
|  | (0.36 - 0.60) | (0.41 - 0.66) | (0.55 - 0.89) | (1.15 - 2.94) | (0.68 - 1.12) | (0.25 - 0.41) | (0.58 - 0.94) | (1.84 - 4.53) | (0.91 - 1.43) | |
| Waiting time (log minutes) | 0.33*** | 0.41*** | 0.36*** | 0.63*** | 1.51*** | 0.93 | 0.89 | 2.08*** | 0.69*** | |
|  | (0.26 - 0.41) | (0.33 - 0.51) | (0.29 - 0.44) | (0.46 - 0.85) | (1.20 - 1.90) | (0.76 - 1.14) | (0.74 - 1.08) | (1.50 - 2.87) | (0.58 - 0.83) | |
| Travel time (log minutes) | 0.76* | 1.31* | 0.67*** | 1.19 | 0.53*** | 1.04 | 0.56*** | 1.32 | 0.54*** | |
|  | (0.57 - 1.03) | (0.98 - 1.75) | (0.51 - 0.88) | (0.74 - 1.93) | (0.38 - 0.75) | (0.75 - 1.46) | (0.41 - 0.77) | (0.76 - 2.31) | (0.40 - 0.74) | |
| OoP expenditure on transport (log córdoba) | 1.09* | 0.95 | 1.11** | 1.17 | 0.84** | 1.26*** | 0.89* | 1.05 | 0.96 | |
|  | (0.98 - 1.21) | (0.86 - 1.05) | (1.01 - 1.23) | (0.96 - 1.43) | (0.73 - 0.97) | (1.09 - 1.45) | (0.79 - 1.00) | (0.82 - 1.34) | (0.85 - 1.08) | |
| OoP expenditure on medicine (log córdoba) | 0.90*** | 0.97 | 0.94** | 0.88*** | 0.75*** | 0.76*** | 0.95 | 0.68*** | 0.91*** | |
|  | (0.84 - 0.95) | (0.92 - 1.03) | (0.89 - 0.99) | (0.79 - 0.96) | (0.70 - 0.81) | (0.71 - 0.81) | (0.89 - 1.02) | (0.61 - 0.77) | (0.86 - 0.97) | |
| Chronic (1-yes) | 0.48*** | 0.52*** | 1.64*** | 0.88 | 0.38*** | 0.46*** | 0.73 | 0.99 | 1.42* | |
|  | (0.32 - 0.72) | (0.35 - 0.76) | (1.14 - 2.37) | (0.45 - 1.70) | (0.25 - 0.59) | (0.30 - 0.71) | (0.49 - 1.11) | (0.49 - 2.01) | (0.95 - 2.11) | |
| 95% CI in parentheses; *** p<0.01, ** p<0.05, * p<0.1.  ^✝^ H3 is a specialist maternity hospital; hence, being female returned unusually large coefficients in this instance. | | | | | | | | | | |

1. Netherlands Enterprise Agency. Construction and Equipment of the General Hospital in Western Managua. 2010. Available from: https://www.rvo.nl/subsidies-regelingen/projecten/construction-and-equipment-general-hospital-western-managua. [↑](#footnote-ref-1)
2. Rao KD, Peters DH, Bandeen-Roche K. Towards patient-centered health services in India - A scale to measure patient perceptions of quality. Int J Qual Heal Care. 2006;18(6):414–21. [↑](#footnote-ref-2)
3. Elkum N, Fahim M, Shoukri M, Al-Madouj A. Which patients wait longer to be seen and when? A waiting time study in the emergency department. East Mediterr Heal J. 2009;15(2):416–24. [↑](#footnote-ref-3)
4. Starkweather J, Moske AK. Multinomial Logistic Regression. 2011. Available from: https://it.unt.edu/sites/default/files/mlr_jds_aug2011.pdf. [↑](#footnote-ref-4)
